# ICNP^®^-Based Nursing Care of a Patient with Erectile Dysfunction, Type 2 Diabetes, and Obesity: A Case Study

**DOI:** 10.3390/reports9020142

**Published:** 2026-05-03

**Authors:** Filip Miłosz Tkaczyk

**Affiliations:** Doctoral School, Collegium Medicum, Jan Kochanowski University of Kielce, 25-369 Kielce, Poland; filip1193@onet.pl; Tel.: +48-791-811-193

**Keywords:** ICNP, nursing care, erectile dysfunction, type 2 diabetes, obesity, case study, chronic disease nursing, sexual health, multimorbidity

## Abstract

Background: Erectile dysfunction (ED) is a common complication of type 2 diabetes and obesity and significantly affects patients’ quality of life. Nursing care for patients with metabolic multimorbidity requires a holistic, structured approach. The International Classification for Nursing Practice (ICNP^®^) enables standardized formulation of nursing diagnoses, interventions, and outcomes and supports structured and individualized ICNP^®^-based care planning. Aim: This study aimed to develop and present an ICNP^®^-based nursing care plan for a patient with erectile dysfunction associated with type 2 diabetes and obesity and to demonstrate the applicability of ICNP^®^ in holistic nursing management of chronic disease. Methods: A descriptive single-case study was conducted in 2025 in a cardiology ward in Poland. Data were collected using a nursing interview, observation, medical documentation analysis, and standardized tools (IIEF-5, SF-36v2). Based on a comprehensive assessment of physical, psychological, and social status, nursing diagnoses, interventions, and expected outcomes were formulated according to ICNP^®^ terminology. Results: The patient presented with poorly controlled diabetes, class I obesity, moderate erectile dysfunction, reduced testosterone levels, and decreased quality of life, particularly in psychosocial domains. Key ICNP^®^ nursing diagnoses included erectile dysfunction, deficient knowledge, obesity, disturbed psychological status, impaired endocrine function, impaired cardiovascular function, and impaired adaptation. Individualized ICNP^®^-based interventions focused on metabolic control, lifestyle modification, sexual health support, education, and psychosocial support. Implementation of the care plan was associated with improvements in health behaviors, disease knowledge, and psychological well-being. Conclusions: ICNP^®^ provides a useful framework for structured and comprehensive nursing care in patients with diabetes-related erectile dysfunction and multimorbidity. Case-based ICNP^®^ care planning supports holistic management, interdisciplinary collaboration, and quality improvement in chronic disease nursing.

## 1. Introduction

Type 2 diabetes is a chronic metabolic disorder characterized by impaired tissue sensitivity to insulin, leading to hyperglycemia. The disease primarily affects adults but is increasingly observed among younger individuals. The main risk factors include obesity, overweight, low physical activity, advanced age, and family history of diabetes [1,2,3,4,5,6]. Erectile dysfunction (ED) is defined as a persistent inability to achieve or maintain an erection sufficient for satisfactory sexual intercourse. It is particularly common among men with type 2 diabetes and has a multifactorial etiology involving vascular, neurological, and psychological mechanisms [7,8,9,10,11,12]. Nursing personnel play a key role in the care of patients with chronic diseases such as diabetes and obesity. Their responsibilities include not only monitoring health parameters but also providing education, emotional support, and assistance in daily disease management [13,14,15,16,17,18].

A holistic approach emphasizes viewing the patient as a whole—considering not only test results but also lifestyle, social relationships, and overall well-being. Nursing staff support patients in developing healthy habits, managing stress, and maintaining physical activity [19,20,21,22,23,24]. Health education involves teaching self-monitoring, recognizing complications, and proper medication use. In cases of intimate issues, such as erectile dysfunction in men with diabetes, nursing personnel should demonstrate empathy, ensure confidentiality, and encourage open communication [25,26,27].

Nursing personnel are essential partners in treatment—helping patients control their disease, strengthen self-esteem, and maintain an active, fulfilling life despite chronic illness [28]. The International Classification for Nursing Practice (ICNP^®^) is a standardized system developed by the International Council of Nurses to provide a unified language for describing nursing diagnoses, interventions, and outcomes. Its use enables precise, evidence-based planning and documentation of the nursing process, enhancing its clarity, consistency, and quality. ICNP^®^ is based on a multi-axial and compositional structure that enables the formulation of nursing diagnoses, interventions, and outcomes through the combination of standardized concepts across different axes (e.g., Focus, Judgment, Action, Means). This approach allows for flexible and context-specific representation of clinical situations, distinguishing ICNP^®^ from more enumerative classification systems. As a result, ICNP^®^ supports both standardization and individualization of nursing care, particularly in complex and multidimensional cases [29,30,31].

Implementing ICNP^®^ in clinical practice and electronic health records promotes standardization of nursing terminology, improves communication within healthcare teams, and allows for comparison of care outcomes at local and international levels. The system also supports evidence-based practice and advances research in nursing [32,33,34]. Despite the widespread use of ICNP^®^ in chronic disease management, there is limited evidence regarding its application in the context of sexual health nursing, particularly in patients with metabolic multimorbidity such as type 2 diabetes and obesity. Erectile dysfunction remains an underrecognized and often insufficiently addressed domain in nursing care, despite its significant impact on quality of life and its association with cardiovascular risk. This case is novel as it integrates ICNP^®^-based nursing care with the assessment and management of sexual dysfunction in a patient with complex metabolic disease, combining physical, psychological, and social dimensions. Additionally, the study incorporates standardized assessment tools (IIEF-5 and SF-36v2) to support a structured and measurable evaluation of patient outcomes.

## 2. Aim of the Study

The aim of this study was to develop an individualized ICNP^®^-based nursing care plan for a patient with erectile dysfunction associated with type 2 diabetes and obesity using the International Classification for Nursing Practice (ICNP^®^).

The author’s objective was to identify key nursing diagnoses, goals, and interventions in holistic care for a patient with multimorbidity, as well as to highlight the role of ICNP^®^ in standardizing documentation and improving the quality of nursing care. The care plan developed in this study was individualized based on the patient’s specific clinical condition, needs, and preferences, while using ICNP^®^ terminology to ensure a structured and standardized framework for documentation.

## 3. Materials and Methods

The study employed the case study method, which allows for a detailed analysis of the nursing care process in a specific clinical situation. The research was conducted in December 2025 in the cardiology ward of a hospital in the Mazovian Voivodeship, after obtaining the patient’s written informed consent to participate and to develop an anonymous case description.

All ethical principles and the provisions of the Declaration of Helsinki were observed. The patient’s data were fully anonymized and used solely for scientific purposes. The study was approved by the Bioethics Committee of the Jan Kochanowski University in Kielce (Decision No. 5/2024 of 19 January 2024).

## 4. Research Method

The case study method was used, allowing for a comprehensive assessment of the patient’s health status, including physical, psychological, and social aspects, as well as the development of an individualized ICNP^®^-based nursing care plan based on the International Classification for Nursing Practice (ICNP^®^). This case report was prepared in accordance with the CARE (CAse REport) guidelines to ensure completeness and transparency of reporting.

## 5. Research Techniques

The following research techniques were applied:

- Nursing interview—used to collect information about the course of the disease, lifestyle, health problems, and the patient’s psychosocial and sexual functioning;
- Observation—both structured and unstructured, focusing on the patient’s behavior, emotional responses, and coping mechanisms related to the illness;
- Analysis of medical documentation—including laboratory test results, specialist consultation reports, and entries from the patient’s medical history.

## 6. Research Tools

To obtain a comprehensive assessment of the patient’s health status and quality of life, the following research tools were used:

- Nursing interview questionnaire—developed based on nursing documentation, allowing the collection of data related to the patient’s physical, psychological, and social condition;
- IIEF-5 scale (International Index of Erectile Function)—used to assess the severity of erectile dysfunction;
- Laboratory test results—including measurements of blood glucose, glycated hemoglobin (HbA1c), lipid profile, testosterone levels, and renal function indicators;
- Penile Doppler ultrasound—performed to evaluate arterial and venous blood flow and identify the underlying causes of erectile dysfunction;
- SF-36v2 scale (Short Form Health Survey)—used to assess the patient’s self-reported quality of life in physical, psychological, and social dimensions. The SF-36v2 questionnaire was selected as a validated and widely used instrument for assessing health-related quality of life across multiple domains, including physical, emotional, and social functioning. Although it is not a disease-specific or strictly patient-centered tool, its multidimensional structure allows for a comprehensive evaluation of patient-perceived health status. In the context of this case, the use of SF-36v2 was complemented by an individualized nursing assessment, enabling a more patient-centered interpretation of quality of life.

## 7. Organization and Course of the Study

The study was conducted in a hospital setting during the patient’s hospitalization and was supplemented with data from outpatient visits. The observation period included five days of inpatient care and an additional analysis of follow-up data.

After obtaining informed consent, a comprehensive assessment of the patient’s condition was performed, covering vital signs, laboratory results, emotional state, health habits, and level of disease-related knowledge. Based on the collected data, nursing diagnoses were formulated in accordance with the ICNP^®^ classification, an individualized ICNP^®^-based nursing care plan was developed, appropriate interventions were implemented, and their effectiveness was evaluated.

## 8. Case Report

Patient W.Z., a 56-year-old man, was admitted in September 2025 to the cardiology ward of a hospital in the Mazovian Voivodeship due to deteriorating general condition, elevated blood glucose levels, and worsening vascular symptoms. The hospitalization was also planned for the purpose of performing coronary angiography.

Medical history revealed type 2 diabetes diagnosed 9 years earlier and progressive erectile dysfunction observed over the past 4 years. The patient was treated with long-acting insulin (insulin glargine—26 units at bedtime) and metformin (2 × 1000 mg). Additionally, class I obesity (BMI = 31.2 kg/m^2^) and arterial hypertension were diagnosed.

The patient had a history of hospitalizations for hypertension and episodes of angina-like chest pain. He did not engage in regular physical activity, led a sedentary lifestyle, and smoked cigarettes (approximately 10 pack-years). During the interview, he expressed low mood, frustration related to erectile dysfunction, and concerns about his relationship with his wife.

## 9. General Condition and Vital Signs on Admission

- General condition: good, fully conscious and oriented
- Skin: dry, without trophic changes
- Heart rate: 84/min, regular
- Blood pressure: 145/90 mmHg
- Body temperature: 36.6 °C
- Oxygen saturation: 98%
- Fasting blood glucose: 162 mg/dL
- HbA1c: 8.4%
- Body weight: 94 kg
- Height: 174 cm
- BMI: 31.2 kg/m^2^

## 10. Laboratory Test Results

Laboratory findings revealed abnormalities typical of poorly controlled type 2 diabetes and dyslipidemia. Fasting glucose was 162 mg/dL, and glycated hemoglobin (HbA1c) was 8.4%, confirming chronic hyperglycemia and inadequate metabolic control. The lipid profile indicated lipid metabolism disorders: total cholesterol 226 mg/dL, LDL 138 mg/dL, HDL 38 mg/dL, and triglycerides 210 mg/dL. These results reflect an increased risk of cardiovascular complications and insufficient lifestyle modification.

The patient also presented with reduced total testosterone levels—9.1 nmol/L (reference range: 10–35 nmol/L), which may have contributed to erectile dysfunction. Renal parameters were within normal limits (creatinine 1.02 mg/dL, eGFR 82 mL/min/1.73 m^2^), indicating preserved kidney function. Overall, the results depict a metabolic profile characteristic of a patient with type 2 diabetes, obesity, and coexisting vascular and hormonal disorders.

## 11. Penile Doppler Ultrasound Results

The results of the Doppler ultrasound examination are presented in Table 1. The findings indicate arterial insufficiency with a component of venous erectile dysfunction, which is characteristic of microangiopathic changes associated with type 2 diabetes.

## 12. Assessment of Erectile Function—IIEF-5 Scale

The patient scored 12 points on the IIEF-5 scale, indicating moderate erectile dysfunction. He reported difficulty maintaining an erection during sexual intercourse and a decreased level of sexual satisfaction.

## 13. Quality of Life Assessment

The patient’s quality of life was evaluated using the SF-36v2 (Short Form Health Survey, License Number: QUO-03978-COQ3T0), which assesses eight domains of physical, psychological, and social functioning. The results indicate a reduced quality of life, particularly in emotional and social aspects.

The patient scored 55 points in physical functioning, reflecting moderate limitations in daily activities. Psychological domains—emotional functioning (40 pts), social functioning (42 pts), and mental health (44 pts)—showed lowered well-being and mood. Overall health was rated at 50 points.

The SF-36v2 profile suggests that comorbid conditions such as diabetes, obesity, and erectile dysfunction significantly affect the patient’s quality of life, emphasizing the need for comprehensive nursing care addressing both physical and psychosocial dimensions.

## 14. Nursing Assessment Results

Based on the interview, observations, and scale evaluations, the patient demonstrated limited knowledge about the impact of lifestyle on sexual health and diabetes management. Reduced motivation to lose weight, difficulties in adhering to a diabetic diet, and low levels of physical activity were also observed. Additionally, the patient exhibited signs of low mood and anxiety, which may affect overall well-being and the effectiveness of therapeutic interventions.

Although long-term follow-up was not available due to the short duration of hospitalization, initial evaluation indicated increased patient engagement in self-care, improved understanding of disease-related mechanisms, and greater openness in discussing sexual health concerns. These observations were supported by structured nursing assessment and patient-reported outcomes collected during hospitalization. The use of standardized tools (IIEF-5 and SF-36v2) enabled baseline quantification of erectile dysfunction severity and quality of life, providing a framework for future outcome monitoring and evaluation of intervention effectiveness.

The patient was not formally consulted by a sexologist or psychologist during hospitalization due to the limited availability of such specialists in the clinical setting. However, psychological aspects, including reduced mood and motivation, were systematically assessed within the nursing process and addressed through patient education, emotional support, and counseling interventions.

## 15. Nursing Care Process Based on the ICNP^®^ Classification

The nursing care process is a fundamental method used by nursing personnel to plan individualized and comprehensive patient care. In the case of a patient with type 2 diabetes, obesity, and erectile dysfunction, it is essential to consider physical, psychological, and social aspects of functioning. The patient’s nursing care process is presented in Table 2.

## 16. Discussion

The presented case illustrates the multifactorial pathophysiology of erectile dysfunction in the context of long-standing type 2 diabetes, obesity, and hypogonadism, which is consistent with current evidence. In our patient, penile Doppler findings indicating arterial insufficiency with a venous component are consistent with diabetes-related microangiopathy. Chronic hyperglycemia contributes to endothelial dysfunction through multiple molecular pathways, including endothelin-1 overexpression, oxidative stress, inflammation, and reduced nitric oxide bioavailability, as demonstrated by Chen et al. [13]. According to the ADA (2026) recommendations, in patients with symptoms of hypogonadism, testosterone levels should also be assessed as part of a comprehensive diagnostic process [35,36]. Furthermore, erectile dysfunction in diabetes should be conceptualized as a neurovascular disorder within the broader spectrum of diabetic neuropathy, involving autonomic and somatic nerve dysfunction, as highlighted by Zamponi et al. [37]. At the cellular level, mechanisms such as apoptosis, autophagy, pyroptosis, and ferroptosis contribute to structural and functional impairment of the corpus cavernosum, as reported by Zhang et al. [38], which may also explain reduced responsiveness to phosphodiesterase type 5 inhibitors in this population. The reduced testosterone level observed in this patient (9.1 nmol/L), together with the IIEF-5 score of 12, indicating moderate erectile dysfunction, is consistent with hypogonadotropic hypogonadism commonly seen in men with obesity and type 2 diabetes; however, evidence suggests that vascular and neuropathic factors often play a more dominant role than hormonal disturbances.

The results of extensive epidemiological analyses confirm the high prevalence of ED in diabetic patients. Kitaw et al., in an umbrella review covering over 100,000 patients, demonstrated that the global prevalence of erectile dysfunction in men with diabetes exceeds 60%, and obesity is an independent risk factor for ED [39]. In addition, the coexistence of reduced mood and impaired quality of life reflects the well-established psychological burden of erectile dysfunction, with depression identified as a significant risk factor in diabetic populations (Dilixiati et al. [40]). Overall, this case supports the need for a holistic and multidisciplinary approach, in which the ICNP^®^ framework enables the integration of physical, psychological, and social dimensions of care, supporting structured and individualized nursing management in complex metabolic disease. The multifactorial nature of the pathogenesis of ED in patients with diabetes was also confirmed in a meta-analysis conducted by Dilixiati et al. The authors indicate that the most significant risk factors include older age, long duration of diabetes, the presence of diabetic neuropathy, cardiovascular disease, hypertension, and depressive symptoms [40].

Ho et al. demonstrated that hormonal disturbances and small fiber neuropathy are associated with erectile dysfunction in men with obesity. Their findings indicate that small fiber neuropathy may play a more important role than hormonal disturbances in this population. These results suggest that damage to neural structures may play a more important role in the pathogenesis of ED than hormonal disorders, particularly in the population of patients with obesity [41]. Regarding therapeutic management, Kamenov emphasizes that phosphodiesterase type 5 inhibitors remain the first-line treatment for men with diabetes, but their effectiveness is significantly lower than in patients without metabolic disorders. 

Contemporary approaches to the care of patients with diabetes, obesity, and erectile dysfunction increasingly rely on models of care for chronic diseases, which emphasize the importance of coordinating the therapeutic team and actively engaging the patient in the treatment process. Glina et al. indicate that effective reduction in risk factors, such as excess body weight, a sedentary lifestyle, and smoking, can significantly improve erectile function and overall metabolic health. In this context, the nurse’s role encompasses not only supporting pharmacotherapy but also systematic health education and motivating the patient to make lasting lifestyle changes. Mulhall et al. point out that patient-centered care, taking into account individual needs, knowledge level, and barriers to self-management, promotes improved quality of life and increases treatment adherence. Furthermore, Redrow et al. emphasize the importance of regular assessment of mental well-being and quality of life, indicating that emotional and psychological support is an essential element of comprehensive care for patients with ED coexisting with metabolic diseases [42,43,44,45].

From a practical perspective, this case represents an example of ICNP^®^ implementation in sexual health nursing in a patient with metabolic multimorbidity. The use of ICNP^®^ enabled structured identification of patient problems, standardized documentation of interventions, and integration of physical, psychological, and social aspects of care, which is consistent with findings from previous studies emphasizing the role of standardized nursing terminologies in improving care organization and communication. Compared to other nursing taxonomies such as NANDA-I and NIC, ICNP^®^ demonstrates distinct structural characteristics; while NANDA-I/NIC may provide more developed diagnostic and intervention frameworks, ICNP^®^ offers greater flexibility through its compositional structure, allowing individualized and context-specific care planning, particularly in complex and sensitive domains such as sexual health (Rabelo-Silva et al.; Müller-Staub et al. [34,46]). These findings align with previous analyses showing that although NANDA-I/NIC systems may achieve higher scores in documentation completeness, ICNP^®^ provides advantages in representing dynamic clinical reasoning processes. Furthermore, its multi-axial structure and compatibility with electronic health records support advanced data processing and interoperability (Hardiker and Rector; Bartz et al. [29,47]).

However, the implementation of ICNP^®^ is associated with important barriers. Studies indicate that limited familiarity with standardized nursing terminologies, insufficient training, and poor integration with electronic health record systems significantly hinder their adoption in clinical practice (Jedwab et al.; Fennelly et al. [48,49]). Moreover, systematic reviews show that the majority of research on ICNP^®^ remains descriptive, with only a small proportion of studies evaluating its impact on patient outcomes (Strudwick and Hardiker; Tastan et al. [50,51]), highlighting a significant evidence gap. In this context, the present case contributes to the existing literature by demonstrating the practical applicability of ICNP^®^ in a complex clinical scenario involving sexual dysfunction, an area often underrepresented in nursing research. Although long-term follow-up data were not available, the observed short-term improvements in patient engagement and communication are consistent with findings from studies indicating that structured, nurse-led interventions and patient education may positively influence health behaviors and self-management in chronic disease populations. Future research should focus on prospective evaluation of ICNP^®^-based interventions using measurable clinical and patient-reported outcomes.

The apparent duplication of certain nursing interventions reflects the multidimensional and iterative nature of the nursing process, in which similar actions may address multiple diagnoses simultaneously. In the present case, patient-directed interventions were organized according to priority clinical needs and grouped into key domains, including metabolic control, sexual health support, psychological well-being, and patient education. This structured approach allowed for coordinated and individualized care while maintaining consistency within the ICNP^®^ framework. Importantly, such organization enhances the practical feasibility of implementing ICNP^®^ in complex clinical settings, particularly in patients with overlapping metabolic and psychosocial conditions.

## 17. Limitations

This study has several limitations that should be acknowledged. First, as a single case report, the findings cannot be generalized to a broader population, and the conclusions should be interpreted with caution. Second, the observation period was limited to the duration of hospitalization, which precluded long-term follow-up and objective assessment of sustained outcomes of the implemented interventions. Third, although standardized tools such as the IIEF-5 and SF-36v2 were used, the evaluation of outcomes relied partially on patient-reported data, which may be subject to response bias. Additionally, the complexity of the patient’s clinical condition, including metabolic, hormonal, and psychological factors, makes it difficult to isolate the specific impact of individual nursing interventions. Finally, the study focuses on the application of ICNP^®^ in a single clinical context, and further research is needed to evaluate its effectiveness in larger populations and different healthcare settings.

## 18. Conclusions

1. A patient with type 2 diabetes, obesity, and erectile dysfunction requires comprehensive, multidisciplinary care that addresses physical, psychological, and social aspects.
2. The use of the ICNP^®^ classification allows for structured and individualized planning, implementation, and evaluation of nursing activities, thereby enhancing the quality and effectiveness of care for patients with chronic diseases.
3. Health education and emotional support play a crucial role in the nursing process, strengthening the patient’s motivation to adopt lifestyle changes, improving glycemic control, and enhancing overall quality of life.
4. Effective collaboration between nursing personnel and the therapeutic team, combined with an individualized approach to the patient, contributes to better treatment outcomes and greater satisfaction with care.
5. This case study confirms that nursing personnel play a key role in health promotion and complication prevention among patients with diabetes and obesity, particularly regarding intimate issues such as erectile dysfunction.

## Figures and Tables

**Table 1:** Hemodynamic parameters of the cavernosal arteries (PSV, EDV, RI).

Parameter	Right Cavernosal Artery	Left Cavernosal Artery	Reference Values
Peak systolic velocity (PSV)	22.4 cm/s	21.9 cm/s	>30 cm/s
End-diastolic velocity (EDV)	5.6 cm/s	5.3 cm/s	<5 cm/s
Resistive index (RI)	0.75	0.76	>0.80

**Table 2:** The Individualized ICNP^®^-Based Nursing Care Process for a Patient with Erectile Dysfunction in the Course of Type 2 Diabetes and Obesity.

ICNP^®^ Diagnosis 1	erectile dysfunction [10009886]
ICNP^®^ Interventions	observation [10013474] assessing sexual functioning [10038706] providing emotional support [10036547] supporting coping [10001741] teaching [10038604] teaching about health condition [10030639] encouraging questions and discussion [10010877] reinforcing adherence to therapeutic regimen [10024562] collaborating with multidisciplinary team [10030809] documenting nursing care [10006173]
ICNP^®^ Result of care	positive reproductive system process [10028156]
ICNP^®^ Diagnosis 2	deficient knowledge [10000837] disease process [10021994]
ICNP^®^ Interventions	observation [10013474] assessing knowledge [10030639] teaching [10038604] teaching about disease [10030639] providing information [10008441] reinforcing adherence to recommendations [10024562] supporting learning [10001741] encouraging questions and discussion [10010877] collaborating with multidisciplinary team [10030809] documenting nursing care [10006173]
ICNP^®^ Result of care	adequate knowledge [10027112]
ICNP^®^ Diagnosis 3	obesity [10013457]
ICNP^®^ Interventions	observation [10013474] assessing nutritional status [10030652] monitoring body weight [10011312] teaching about diet regimen [10021939] teaching about healthy lifestyle [10036447] teaching about physical activity [10022585] promoting physical activity [10007315] assisting with lifestyle modification [10024734] reinforcing adherence to recommendations [10024562] collaborating with multidisciplinary team [10030809] documenting nursing care [10006173]
ICNP^®^ Result of care	adequate nutritional status [10042065] positive metabolic process [10028156] positive health behavior [10029134] improved quality of life [10028194]
ICNP^®^ Diagnosis 4	disturbed psychological status [10038411]
ICNP^®^ Interventions	observation [10013474] providing emotional support [10036547] encouraging rest [10041415] collaborating with multidisciplinary team [10030809] documenting nursing care [10006173]
ICNP^®^ Result of care	positive psychological status [10040670]
ICNP^®^ Diagnosis 5	impaired endocrine system function [10022965]
ICNP^®^ Interventions	observation [10013474] monitoring blood glucose level [10032020] administering insulin [10030417] teaching about disease [10030639] teaching about medication regimen [10021941] teaching about diet regimen [10021939] reinforcing adherence to therapeutic regimen [10024562] collaborating with multidisciplinary team [10030809] documenting nursing care [10006173]
ICNP^®^ Result of care	positive physiological process [10028156]
ICNP^®^ Diagnosis 6	impaired cardiovascular system function [10022949]
ICNP^®^ Interventions	observation [10013474] monitoring blood pressure [10003335] monitoring heart rate [10008833] assessing cardiovascular status [10002887] administering medication [10025444] promoting rest [10041415] teaching about disease [10030639] teaching about lifestyle modification [10036447] reinforcing adherence to therapeutic regimen [10024562] collaborating with multidisciplinary team [10030809] documenting nursing care [10006173]
ICNP^®^ Result of care	positive cardiovascular system process [10028102]
ICNP^®^ Diagnosis 7	impaired adaptation [10022027]
ICNP^®^ Interventions	observation [10013474] providing emotional support [10036547] supporting coping [10001741] encouraging rest [10041415] teaching about disease [10030639] reinforcing adherence to therapeutic regimen [10024562] collaborating with multidisciplinary team [10030809] documenting nursing care [10006173]
ICNP^®^ Result of care	positive health behavior [10029134]

## Data Availability

The data are not publicly available due to privacy and ethical restrictions.
